# ENDOGENOUS DNA DAMAGE AS A DRIVER OF AGING AND TISSUE DYSFUNCTION

**DOI:** 10.1093/geroni/igac059.783

**Published:** 2022-12-20

**Authors:** Matt Yousefzadeh

**Affiliations:** University of Minnesota, Minneapolis, Minnesota, United States

## Abstract

Aging is a complex multifactorial process that enhances stress or impairs the ability to cope with it, whereby increasing the risk of morbidity and mortality. Many factors can contribute to aging, such as macromolecular damage (including DNA damage) that accumulates in a time-dependent manner. DNA damage is also known to induce cellular senescence, a cell fate that is known to play a causal role in aging. To investigate the effects of DNA damage and cellular senescence on aging, animal models lacking Ercc1, an important DNA repair gene, were utilized. Ercc1-deficient mice age faster than wild-type littermates and exhibit the senescent cell burdens that are comparable to that of a naturally aged mouse. To investigate cell autonomous and non-autonomous effects of DNA repair deficiency on senescence and aging, Ercc1 was deleted in mice in a tissue-specific manner. Increased senescent cell burden and dysfunction were present in tissues specifically targeted for Ercc1 deletion. However, enhanced senescence was also present in some non-targeted tissues, suggesting that these occur through cell non-autonomous mechanisms.

